# Intelligent Staging Performance of Diabetic Retinopathy Based on Fundus Fluorescein Angiography Images with Different Angiographic Phases

**DOI:** 10.3390/bioengineering13070791

**Published:** 2026-07-10

**Authors:** Wei Wang, Zhenpeng Chen, Mingming Li, Shuang Li, Kang Wang, Haiyun Li

**Affiliations:** 1 Beijing Key Laboratory of Clinical Engineering Solutions for Mental Health, School of Biomedical Engineering, Capital Medical University, Beijing 100069, China; wangwei8542@126.com (W.W.); 18366108084@163.com (Z.C.); 2 Department of Ophthalmology, Beijing Friendship Hospital, Capital Medical University, Beijing 100050, China; yyyy_mingmingli@126.com (M.L.); lishuang8023@ccmu.edu.cn (S.L.); bnbn2000@163.com (K.W.)

**Keywords:** diabetic retinopathy, fluorescein fundus angiography, Swin Transformer, ConvNeXt, angiographic phase, grading model

## Abstract

Fundus fluorescein angiography (FFA) is the gold standard for diabetic retinopathy (DR) staging, yet whether angiographic phase affects deep learning performance remains unknown. Using 7508 FFA images from 863 eyes, stratified into venous, recirculation, and late phases, we developed Swin Transformer- and ConvNeXt-based DR staging models under the International five-grade and Chinese six-grade classification systems. Multiclass and binary (NPDR vs. PDR) classification tasks were evaluated. This study provides the first systematic quantification of angiographic phase effects on FFA-based DR staging. Phase-related performance was analyzed using generalized linear mixed-effects models with beta regression. ConvNeXt generally outperformed Swin Transformer, particularly in multiclass classification. Across all experimental settings, performance showed a consistent numerical decline from the venous to the late phase. Under the International five-grade system, ConvNeXt accuracy declined from 86.67% to 81.87%; however, Bonferroni-adjusted comparisons revealed no statistically significant phase-related differences (all adjusted *p* > 0.05), with small effect sizes (|SMD| < 0.2). Binary classification remained highly stable across phases, with accuracies exceeding 91%. Grad-CAM visualizations demonstrated progressively diffuse model attention in later phases. These findings support phase-flexible FFA acquisition for binary DR screening, whereas venous- or recirculation-phase images remain preferable for high-precision multiclass staging, guiding phase-aware AI development.

## 1. Introduction

Diabetic retinopathy (DR) is a leading cause of visual impairment and blindness, affecting approximately one-third of individuals with diabetes [1,2,3,4,5]. Vision loss mainly results from diabetic macular edema (DME) and proliferative diabetic retinopathy (PDR) [6,7,8]. Early diagnosis and timely intervention play a vital role in preventing vision loss. Therefore, accurate DR staging is essential for assessing disease severity, guiding treatment decisions, and monitoring disease progression [9,10].

Fundus fluorescein angiography (FFA) is the gold standard for in vivo visualization of the retinal vasculature [11]. FFA accurately delineates lesions associated with different DR stages, including microaneurysms, exudates, non-perfused areas, intraretinal microvascular abnormalities (IRMA), neovascularization, vascular leakage, and macular edema [12]. Moreover, as a dynamic imaging modality, FFA traces fluorescein transit through the retinal vasculature over time, generating phase-specific images with distinct imaging characteristics [13,14].

Deep learning models have been widely applied to FFA-based DR staging [15]. The Ai-Doctor system first demonstrated that deep learning can directly perform DR staging from FFA images, achieving an AUC of 0.991–0.999 [16]. The Inter-preFFA framework improved the extraction of dynamic retinal pathological features via contrastive learning [17]. The LLM-assisted system explored interactive report generation [18]. Rasta et al. proposed an unsupervised algorithm for detecting non-perfusion areas, achieving 81% sensitivity, 78% specificity, and 91% accuracy [19]. Gao et al. developed a deep learning system using a large-scale FFA dataset, with VGG16 achieving a maximum accuracy of 94.17% [20]. Pan et al. developed a multi-label model detecting four lesion types (non-perfusion, microaneurysms, leakage, laser scars), with DenseNet achieving AUCs of 0.8703, 0.9435, 0.9647, and 0.9653, respectively [21]. Despite these advances, the existing literature shares a common limitation: nearly all studies treat FFA images as static inputs, focusing primarily on architectural improvements to improve classification performance on fixed-image datasets. While this approach has driven substantial progress, it does not explicitly consider an important clinical characteristic of FFA—namely, that angiography is inherently a time-resolved dynamic process. During a standard FFA examination, fluorescein circulates through the retinal vasculature over time, producing phase-specific images with markedly different lesion visibility. Venous phase images provide high vascular contrast and clear visualization of retinal lesions, whereas progressive dye leakage during later phases reduces vessel-to-background contrast and may partially or completely obscure key pathological features, including microaneurysms and hemorrhages [22,23].

Theoretically, these phase-dependent image variations could substantially affect deep learning model performance, potentially leading to two challenges in late phase images: (i) diminished contrast may impede the identification of fine microvascular details, and (ii) extensive dye leakage may obscure key pathological features, increasing the risk of false-negative predictions. However, to date, no study has systematically quantified the impact of angiographic phase on DR staging accuracy, nor has any study rigorously tested whether performance differences across angiographic phases are statistically significant. This knowledge gap is particularly relevant in real-world clinical settings, where FFA image acquisition timing varies with individual patient circulatory dynamics and operator protocols. If model performance is indeed sensitive to angiographic phase, current static-image benchmarks may not fully reflect real-world performance, potentially leading to misguided deployment decisions.

Accordingly, this study addresses these knowledge gaps through three complementary objectives. First, by adopting both the International five-grade and Chinese six-grade classification systems, we evaluate model generalization across different clinical grading frameworks. Second, we provide the first systematic quantification of the effect of angiographic phase on DR staging performance using GLMMs with beta regression and nested random effects, while reporting 95% confidence intervals, effect sizes, and Bonferroni-adjusted *p* values. Third, by comparing Swin Transformer and ConvNeXt, we explore the relative merits of attention-based versus convolution-based architectures in handling phase-induced image variations. The findings of this study are expected to provide actionable insights for clinical practice. If phase-related performance differences are minimal, FFA acquisition protocols may be relaxed to reduce operational burden. In addition, phase metadata could potentially facilitate phase-aware confidence calibration in future AI systems, although this strategy requires prospective evaluation.

## 2. Materials and Methods

A total of 7508 FFA images from patients with diabetic retinopathy (DR) were included in this study. All images were independently annotated according to both the International five-grade and Chinese six-grade DR classification systems. Annotation discrepancies were resolved by consensus to ensure label accuracy. FFA images were stratified into three angiographic-phase groups: venous, recirculation, and late phases. Swin Transformer and ConvNeXt architectures were used to develop DR staging models. Both multiclass and binary classification tasks were performed under each grading system. Model performance was evaluated using accuracy, precision, recall, and F1-score. Statistical analyses were performed using generalized linear mixed-effects models (GLMMs) with beta regression to assess the effect of angiographic phase on model performance.

### 2.1. Sample Collection

Patients who underwent fundus fluorescein angiography (FFA) for the evaluation of diabetic retinopathy (DR) at Beijing Friendship Hospital between May 2016 and December 2025 were retrospectively screened. All FFA images were acquired using a Heidelberg Spectralis HRA angiography system (Heidelberg Engineering, Heidelberg, Germany). The study was approved by the institutional ethics committee. A total of 450 patients (863 eyes; mean age, 63.8 ± 12.6 years; male-to-female ratio, 221:229) were included. The inclusion criteria were as follows: (1) FFA images obtained from patients with newly diagnosed diabetic retinopathy. (2) FFA images acquired during the venous, recirculation, or late angiographic phases. The exclusion criteria were as follows: (1) Poor-quality images caused by ocular diseases other than DR. (2) Images from eyes that had undergone any previous retinal treatment. (3) FFA images acquired before the venous phase. After applying these criteria, a total of 7508 FFA images were included in the final dataset. To prevent data leakage, the dataset was randomly partitioned into training and testing sets (8:2) at the eye level, ensuring that all images from each eye were assigned exclusively to a single partition. Specifically, the 863 eyes were allocated to a training set (690 eyes, 80%) and a test set (173 eyes, 20%). For each eye, all images acquired during the venous, recirculation, and late phases were assigned to the same partition. This strategy prevented data leakage while ensuring complete independence between the training and testing sets.

### 2.2. DR Grading Standards and Image Annotation

FFA images were independently annotated according to both the International five-grade and Chinese six-grade DR grading systems. The International five-grade clinical classification system categorizes DR into five severity categories: no apparent diabetic retinopathy, mild non-proliferative diabetic retinopathy (NPDR), moderate NPDR, severe NPDR, and proliferative diabetic retinopathy (PDR). The Chinese six-grade classification system was established by the Fundus Disease Study Group of the Ophthalmology Branch of the Chinese Medical Association in 2014. It further refines the proliferative stage and defines six stages: microaneurysm stage, mild-to-moderate NPDR stage, severe NPDR stage, early PDR stage, moderate- and high-risk PDR stage, and advanced decompensated PDR stage. The Chinese six-grade dataset comprised fewer images (6717 vs. 7508) because eyes classified as “no apparent diabetic retinopathy” under the International system were excluded.

All images were independently graded by three retinal specialists from Beijing Friendship Hospital, Capital Medical University, each with more than 10 years of experience in retinal disease diagnosis. Before annotation, all annotators received standardized training to harmonize the diagnostic criteria and key differential diagnostic features across the two grading systems, thereby minimizing inter-observer variability. When disagreements occurred, a senior retinal specialist adjudicated the discrepancies by consensus to determine the final label. Inter-rater reliability among the three annotators was evaluated using Fleiss’ kappa, which yielded values of 0.86 and 0.83 for the International five-grade and Chinese six-grade standards, respectively, with corresponding percent agreements of 91.2% and 88.7%. These results indicate almost perfect agreement (κ > 0.81), supporting the reliability of the reference standard labels used for model training. The sample distributions under the two grading systems are presented in Table 1 and Table 2. For the binary classification task, DR was further categorized as NPDR or PDR. To minimize potential bias introduced by class imbalance, the two classes were balanced to include 1427 images each, yielding a total of 2854 images (Table 3).

### 2.3. Sample Grouping

Before phase-wise grouping, eye-level partitioning was first performed to prevent data leakage between the training and testing sets. According to the temporal sequence of FFA imaging, all included FFA images were categorized into three non-overlapping angiographic phases: venous phase (V, 25 s–2 min after contrast injection), recirculation phase (R, 2.5–6 min), and late phase (L, 7–10 min) [24]. The three phase groups were mutually exclusive. Image distributions under the two DR classification systems are summarized in Table 4 and Table 5. The binary classification task used the same phase-grouping strategy, with the corresponding image distribution summarized in Table 6.

### 2.4. Deep Learning Models for FFA-Based DR Staging

Two deep learning architectures, Swin Transformer and ConvNeXt, were used to develop DR staging models based on FFA images.

#### 2.4.1. Swin Transformer-Based DR Staging Model

**Figure 1:**
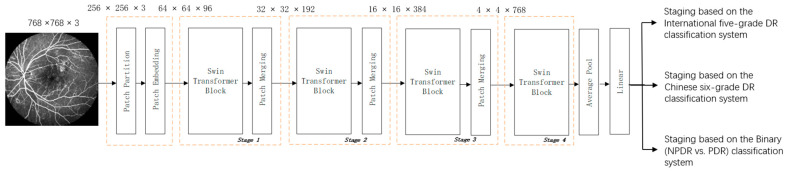
Overview of the Swin-Tiny architecture.

The Swin Transformer served as the backbone network for DR staging (Figure 1). Swin Transformer employs a shifted-window self-attention mechanism that reduces computational complexity while enabling information exchange between adjacent windows. The Swin-Tiny (Swin-T) variant was employed. The model comprised four stages with a window size of 7 × 7, a head dimension of 32, and a multilayer perceptron (MLP) expansion ratio of 4. Transfer learning was applied using pre-trained weights from ImageNet-22K, where the parameters of the first two stages were frozen, and the last two stages were fine-tuned to adapt to the distinctive features of FFA images.

#### 2.4.2. ConvNeXt-Based DR Staging Model

**Figure 2:**
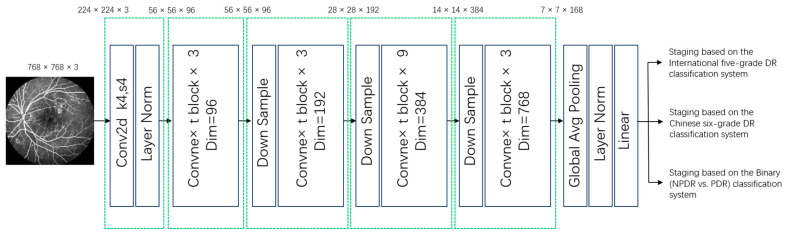
Overview of the ConvNeXt architecture.

ConvNeXt was adopted as the backbone network for DR staging, as shown in Figure 2. ConvNeXt is a convolutional neural network architecture redesigned using several principles inspired by Vision Transformers. In this study, the ConvNeXt-Tiny variant was adopted. Its key architectural features include a patchify stem layer, depthwise separable convolutions, an inverted bottleneck structure, large-kernel depthwise convolutions (7 × 7), and a reduced number of activation and normalization layers. Transfer learning was applied using pre-trained weights from ImageNet-22K, where the parameters of the first two stages were frozen, and the last two stages were fine-tuned to adapt to the distinctive features of FFA images.

The two architectures were compared to evaluate the relative performance of attention-based and convolution-based approaches for FFA-based DR staging. For both architectures, identical training configurations were adopted. Input images were resized to 256 × 256 for Swin Transformer and 224 × 224 for ConvNeXt, consistent with the default settings of each architecture. Both models were optimized using the AdamW optimizer with an initial learning rate of 1 × 10^−4^ and a weight decay of 1 × 10^−5^. The batch size was set to 32, and each model was trained for 100 epochs. Each experiment was independently repeated three times to improve the robustness of the results. No data augmentation was applied, thereby preserving the original image distribution throughout model training. For the binary classification tasks, the NPDR and PDR classes were balanced to include 1427 images each. Model performance was evaluated exclusively on the independent test set, which remained completely unseen during model training.

Experiments were conducted on Ubuntu (Canonical Ltd., London, UK) using Python 3.8 (Python Software Foundation, Wilmington, DE, USA) and a single NVIDIA RTX 3090 GPU (24 GB memory). Cross-entropy loss was used for both multiclass and binary classification tasks.

### 2.5. Statistical Analysis

Because the evaluated performance metrics were expressed as proportions bounded between 0 and 1, generalized linear mixed-effects models (GLMMs) with beta regression were fitted using R software version 4.6.0 (R Foundation for Statistical Computing, Vienna, Austria). The generalized linear mixed models were fitted using the glmmTMB package. Angiographic phase, eye side, and fundus orientation were entered as fixed effects. Three nested random-effects structures were compared using the Akaike information criterion (AIC) and Bayesian information criterion (BIC): (1) random intercept for patient ID only [(1|patient ID)]; (2) random intercept for patient ID with eye side nested within patient ID [(1|patient ID/eye side)]; and (3) random intercept for patient ID with eye side nested within patient ID and fundus orientation nested within patient ID/eye side [(1|patient ID/eye side/fundus orientation)]. The random-intercept model including patient ID only yielded the lowest AIC and BIC values and was therefore selected as the final model for all subsequent analyses, providing the most parsimonious fit while appropriately accounting for correlations among repeated observations from the same patient. Pairwise comparisons among angiographic phases were performed using estimated marginal means (EMMs) with Bonferroni adjustment.

### 2.6. Grad-CAM Interpretability Analysis

To improve model interpretability, Gradient-weighted Class Activation Mapping (Grad-CAM) was applied to all trained models under both DR classification systems. Representative FFA images covering different DR stages and the venous, recirculation, and late angiographic phases were selected for visualization. For each input image, the gradients of the predicted class score were backpropagated to the final feature maps to generate class-discriminative heatmaps. The resulting heatmaps were overlaid onto the original FFA images to highlight regions contributing most to the model predictions, enabling qualitative comparison of model attention across different angiographic phases. Grad-CAM analyses were performed for all trained models; for brevity, only representative visualizations from the best-performing ConvNeXt model trained under the International five-grade DR classification system are presented in the main text.

## 3. Results

Model performance is presented separately for the International five-grade DR classification system, the Chinese six-grade DR classification system, and binary classification (NPDR vs. PDR), with comparisons across the venous (V), recirculation (R), and late (L) angiographic phases. Performance was assessed using accuracy, precision, recall, and F1-score. Statistical comparisons based on generalized linear mixed-effects models (GLMMs) with beta regression are reported together with Bonferroni-adjusted pairwise comparisons and standardized mean differences (SMDs). Grad-CAM analyses were further performed to visualize model attention across angiographic phases.

### 3.1. Performance Under the International Five-Grade DR Classification System

Under the International five-grade standard, ConvNeXt consistently outperformed Swin Transformer across all phase groups (Table 7). In the venous phase (V), ConvNeXt achieved an accuracy of 86.67%, precision of 84.62%, recall of 85.26%, and F1-score of 84.91%, compared with 83.55%, 82.15%, 80.12%, and 80.83%, respectively, for Swin Transformer. Both models exhibited a gradual numerical decline with increasing angiographic phase. Swin Transformer accuracy declined from 83.55% (V) to 77.46% (L), whereas ConvNeXt declined from 86.67% to 81.87%. Similar trends were observed across all metrics.

GLMM results are summarized in Table 8. Model selection via AIC/BIC favored the simplest random-intercept structure (1|patient ID) for both architectures (Supplementary Table S1), and the corresponding parameter estimates are presented in Table 8. Estimated marginal probabilities ranged from 0.796 to 0.809 for Swin Transformer and 0.895 to 0.910 for ConvNeXt across phases (Supplementary Table S2). Bonferroni-corrected pairwise comparisons revealed no statistically significant differences between any phase groups (all adjusted *p* > 0.05; Table 9), with consistently small effect sizes (|SMD| < 0.2 for all contrasts).

### 3.2. Performance Under the Chinese Six-Grade DR Classification System

Under the Chinese six-grade standard, ConvNeXt again outperformed Swin Transformer across all phase groups (Table 10). In the venous phase (V), ConvNeXt achieved an accuracy of 85.78%, precision of 89.57%, recall of 86.60%, and F1-score of 87.77%, compared with 80.87%, 86.00%, 77.37%, and 80.97%, respectively, for Swin Transformer. Both models exhibited a gradual numerical decline with increasing angiographic phase. Swin Transformer accuracy declined from 80.87% (V) to 76.66% (L), whereas ConvNeXt declined from 85.78% to 80.98%. Comparable trends were observed across all metrics.

GLMM results are summarized in Table 11. Model selection via AIC/BIC favored the simplest random-intercept structure (1|patient ID) for both architectures (Supplementary Table S1), and the corresponding parameter estimates are presented in Table 11. Estimated marginal probabilities ranged from 0.788 to 0.797 for Swin Transformer and 0.900 to 0.909 for ConvNeXt across phases (Supplementary Table S2). Bonferroni-corrected pairwise comparisons revealed no statistically significant differences between any phase groups (all adjusted *p* > 0.05; Table 12), with consistently small effect sizes (|SMD| < 0.2 for all contrasts).

### 3.3. Performance Under the International Binary (NPDR vs. PDR) Classification System

Under the International binary classification standard (NPDR vs. PDR), ConvNeXt consistently outperformed Swin Transformer across all phase groups (Table 13). In the venous phase (V), ConvNeXt achieved an accuracy of 94.92%, precision of 95.17%, recall of 89.32%, and F1-score of 91.86%, compared with 93.75%, 91.57%, 89.26%, and 90.35%, respectively, for Swin Transformer. Both models exhibited only slight numerical declines with increasing angiographic phase. Swin Transformer accuracy declined from 93.75% (V) to 92.76% (L), whereas ConvNeXt declined from 94.92% to 94.06%. The remaining performance metrics followed a similar pattern.

GLMM results are summarized in Table 14. Model selection via AIC/BIC favored the simplest random-intercept structure (1|patient ID) for both architectures (Supplementary Table S1), and the corresponding parameter estimates are presented in Table 14. Estimated marginal probabilities ranged from 0.897 to 0.906 for Swin Transformer and 0.950 to 0.958 for ConvNeXt across phases (Supplementary Table S2). Bonferroni-corrected pairwise comparisons revealed no statistically significant differences between any phase groups (all adjusted *p* > 0.05; Table 15), with consistently small effect sizes (|SMD| < 0.2 for all contrasts).

### 3.4. Performance Under the Chinese Binary (NPDR vs. PDR) Classification Systems

Under the Chinese binary classification standard (NPDR vs. PDR), ConvNeXt again achieved comparable or slightly better performance than Swin Transformer across all angiographic phases (Table 16). In the venous phase (V), ConvNeXt achieved an accuracy of 93.30%, precision of 93.86%, recall of 93.30%, and F1-score of 93.28%, compared with 93.30%, 93.41%, 93.30%, and 93.29%, respectively, for Swin Transformer. Both architectures demonstrated highly stable performance across the venous, recirculation, and late phases, with only minimal numerical variation in all evaluation metrics. Accuracy remained above 91% for both models throughout all phase groups, while precision, recall, and F1-score exhibited similarly consistent performance.

GLMM results are summarized in Table 17. Model selection based on AIC and BIC again favored the simplest random-intercept structure (1|patient ID) for both architectures (Supplementary Table S1), and the corresponding parameter estimates are presented in Table 17. Estimated marginal probabilities ranged from 0.878 to 0.891 for Swin Transformer and 0.935 to 0.948 for ConvNeXt across the three angiographic phases (Supplementary Table S2), further supporting the high phase robustness of binary classification. Bonferroni-corrected pairwise comparisons revealed no statistically significant differences between any phase groups (all adjusted *p* > 0.05; Table 18), with consistently small effect sizes (|SMD| < 0.2 for all contrasts).

### 3.5. Grad-CAM Visualization of Model Attention

**Figure 3:**
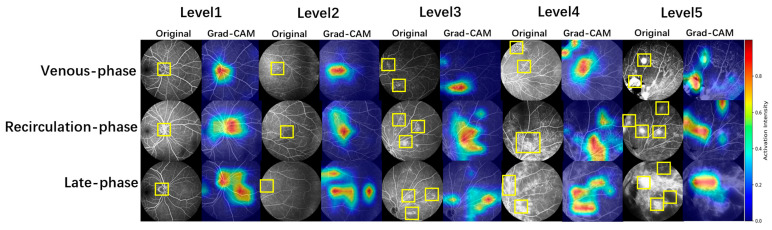
Grad-CAM heatmaps of representative FFA images across different phases using ConvNeXt. Each row shows venous, recirculation, and late phase original images (left) and their Grad-CAM overlays (right) for representative DR stages (Levels 1–5, as labeled in each panel), with representative lesions marked by yellow boxes. Red-to-blue colormap indicates decreasing model attention. From top to bottom, model attention shifts from precise vascular/lesion localization in the venous phase, to progressive diffusion in the recirculation phase, and finally to extensive leakage areas away from small lesions (e.g., microaneurysms) in the late phase.

Grad-CAM analyses were performed for all trained models. Because similar phase-dependent attention patterns were observed across architectures and classification systems, only representative visualizations from the ConvNeXt model trained under the International five-grade DR classification system are presented (Figure 3).

In the venous phase (first row), model attention was primarily concentrated on retinal structures, including vascular abnormalities and hyperfluorescent lesions. In the recirculation phase (second row), attention maps became more dispersed as fluorescein leakage increased. In the late phase (third row), attention became markedly more diffuse, shifting from focal lesions (e.g., microaneurysms) toward extensive leakage areas.

A progressive shift in model attention from focal lesions toward diffuse leakage regions was observed across angiographic phases, consistent with the gradual numerical performance decline reported in Table 7, Table 10, Table 13 and Table 16.

## 4. Discussion

The present study provides the first systematic quantification of the impact of angiographic phase on FFA-based deep learning DR staging. Across two architectures, two grading standards, and multiple classification tasks, we observed a consistent downward trend in classification performance from the venous to the late angiographic phase. However, GLMM-based testing with Bonferroni correction revealed no statistically significant phase-related differences (all adjusted *p* > 0.05), with predominantly small effect sizes (|SMD| < 0.2). Overall classification performance remained comparable to that reported in previous FFA-based DR studies (Supplementary Table S3), with multiclass and binary classification accuracies falling within the ranges previously reported in the literature (79.2–94.17% and 90.0–95.8%, respectively). Importantly, beyond achieving comparable classification performance, the present study extends previous work by providing the first systematic evaluation of how angiographic phase influences FFA-based deep learning DR staging across different grading systems, model architectures, and classification tasks. These findings suggest that angiographic phase exerts a subtle rather than decisive influence on model performance and provide a basis for exploring the underlying mechanisms and their clinical implications.

The consistent numerical decline observed from the venous to the late angiographic phase is likely driven, at least in part, by progressive changes in FFA image characteristics during angiography rather than solely by intrinsic limitations of the evaluated deep learning models. As angiography progresses into the late phase, vessel-to-background contrast gradually decreases, reducing the conspicuity of fine vascular abnormalities such as microaneurysms, intraretinal microvascular abnormalities (IRMA), and capillary non-perfusion areas [25]. Simultaneously, progressive hyperfluorescent leakage—particularly prominent in proliferative diabetic retinopathy owing to immature neovascular endothelium—can partially or completely obscure focal pathological lesions [26,27], thereby reducing the discriminability of image features available for automated classification. This interpretation is further supported by the Grad-CAM visualizations (Figure 3), which provide qualitative evidence that model attention progressively shifts from discrete vascular lesions in the venous phase toward diffuse leakage regions in the late phase, paralleling the observed numerical decline in classification performance. Nevertheless, despite these plausible imaging changes, none of the observed performance differences reached statistical significance after GLMM analysis with Bonferroni correction. These findings suggest that the evaluated deep learning architectures are relatively robust to the degree of image variability introduced by routine angiographic phase variation, although subtle effects on feature representation may still contribute to the consistent numerical performance trend observed across phases.

Although both architectures exhibited a numerical decline in performance from the venous to the late angiographic phase, the magnitude of this decline was consistently smaller for ConvNeXt, which generally outperformed Swin Transformer across most experimental settings, particularly in the multiclass classification tasks. These findings suggest that model architecture may exert a greater influence on FFA-based DR staging performance than routine angiographic phase variation. This superior performance may be related to the convolution-based inductive biases of ConvNeXt, which are generally considered advantageous for learning local spatial patterns and maintaining stable feature representations under varying image contrast conditions. In contrast, Swin Transformer exhibited a greater numerical performance decline across angiographic phases, which may reflect increased sensitivity to the reduced vessel-to-background contrast and diffuse leakage observed in later phases. However, these interpretations should be considered hypothesis-generating rather than conclusive, as the present study did not include direct analyses of feature representations or attention mechanisms.

Beyond the overall phase trend and architectural differences, the clinical impact of angiographic phase was largely task-dependent. Binary NPDR-versus-PDR classification remained highly stable across all angiographic phases, indicating that this classification task was relatively less affected by routine phase-related image variation. Unlike fine-grained multiclass staging, binary classification primarily depends on identifying the presence or absence of neovascularization rather than subtle lesion characterization. Accordingly, this robustness is clinically plausible because the defining feature of PDR—neovascularization—produces progressive hyperfluorescent leakage that becomes increasingly conspicuous during later angiographic phases, thereby providing the model with an amplifying rather than diminishing diagnostic signal. In contrast, fine-grained multiclass staging showed substantially greater sensitivity to angiographic phase. Accurate discrimination between adjacent DR severity levels relies on detecting subtle retinal lesions, such as microaneurysms, intraretinal microvascular abnormalities, and localized capillary non-perfusion areas, all of which become progressively less conspicuous as fluorescein leakage increases. This task-dependent pattern was most evident in the Chinese six-grade classification system, where the numerical decline across angiographic phases was greater than that observed for binary classification, although statistical significance was not reached. These findings therefore indicate that the influence of angiographic phase should be interpreted in the context of the intended clinical application, with venous and recirculation phase images being potentially advantageous for high-precision multiclass DR staging.

Beyond their mechanistic interpretation, these findings have several practical implications for both clinical FFA acquisition and the future development of phase-aware AI systems. First, for binary screening (NPDR vs. PDR), where phase-related performance differences appear minimal, FFA images acquired during any routine angiographic phase are likely to support reliable AI-assisted screening performance, potentially reducing the need for strict acquisition timing in busy clinical practice. Second, for developing high-precision fine-grained DR staging models, venous- or recirculation-phase images may represent the preferred image source, given their superior lesion conspicuity and vessel-to-background contrast. The highly comparable performance of Swin Transformer and ConvNeXt under the Chinese binary classification system further supports the robustness of binary DR screening across both model architectures. Because angiographic phase introduces subtle but consistent image variability without substantially affecting overall classification performance, explicit incorporation of phase information may improve model reliability rather than classification accuracy. Accordingly, phase metadata could potentially be incorporated into future AI systems for confidence calibration or uncertainty estimation, although this concept remains hypothetical and warrants prospective validation before clinical implementation. Future studies should further investigate whether incorporating images from multiple angiographic phases into model training, or adopting explicit phase-aware learning strategies, can improve cross-phase generalization and model robustness.

Several limitations of this study should be acknowledged. First, this was a retrospective single-center study conducted in a Chinese patient population, and the generalizability of our findings requires validation in multicenter external cohorts. Second, although angiographic phases were defined using standardized acquisition time windows, individual variations in circulation dynamics may introduce minor variability in phase assignment. Third, class imbalance in the advanced stages of the Chinese six-grade classification system may have contributed to reduced recall in minority classes, although per-class performance metrics were provided to facilitate transparent evaluation (Supplementary Tables S4–S9). Fourth, only two representative deep learning architectures, ConvNeXt and Swin Transformer, were evaluated; whether the observed phase-related trends extend to other architectures remains to be determined. In addition, all FFA images were acquired using a single Heidelberg Spectralis HRA system, and differences among FFA imaging platforms may influence image characteristics and model performance. Finally, although no statistically significant phase-related differences were detected, subtle phase-related effects may become detectable in larger multicenter datasets with greater statistical power. Future studies should therefore incorporate external validation cohorts, multiple imaging platforms, and diverse model architectures to further evaluate the robustness and clinical generalizability of FFA-based DR staging across different angiographic phases.

## 5. Conclusions

This study provides the first systematic quantification of how angiographic phase influences FFA-based deep learning models for diabetic retinopathy staging. Across different model architectures, grading standards, and classification tasks, venous-phase images consistently yielded the highest classification performance, while ConvNeXt generally achieved the best overall performance, particularly in multiclass classification. Although classification performance showed a consistent numerical decline from the venous to the late angiographic phase across all experimental settings, rigorous GLMM-based statistical testing with Bonferroni correction demonstrated no statistically significant phase-related differences (all corrected *p* > 0.05), with predominantly small effect sizes (|SMD| < 0.2). These findings suggest that angiographic phase should be considered as a practical optimization factor rather than a major determinant of model performance. Clinically, phase-flexible FFA acquisition appears sufficient for binary DR screening (NPDR vs. PDR), whereas venous- or recirculation-phase images remain preferable for high-precision fine-grained DR staging. Future multicenter prospective studies incorporating external validation cohorts, multiple imaging platforms, and diverse deep learning architectures are warranted to further evaluate phase-aware AI strategies and improve model robustness and clinical generalizability.

## Figures and Tables

**Table 1:** Distribution of images under the International five-grade DR classification system.

Dataset	Level 1	Level 2	Level 3	Level 4	Level 5	Total
Sample size	791	635	1353	3222	1507	7508
Percentage	10.54%	8.46%	18.02%	42.91%	20.07%	100%

**Table 2:** Distribution of images under the Chinese six-grade DR classification system.

Dataset	Level 1	Level 2	Level 3	Level 4	Level 5	Level 6	Total
Sample size	635	1353	3222	1013	303	191	6717
Percentage	9.45%	20.14%	47.97%	15.08%	4.51%	2.84%	100%

**Table 3:** Distribution of images in the binary DR classification system.

Dataset	International			China		
	NPDR	PDR	Total	NPDR	PDR	Total
Sample size	1427	1427	2854	1427	1427	2854
Percentage	50%	50%	100%	50%	50%	100%

**Table 4:** Distribution of images across angiographic phases under the International five-grade DR classification system.

Phase Group	Level 1	Level 2	Level 3	Level 4	Level 5	Total
V	263	207	465	942	487	2364
R	300	293	646	1709	785	3733
L	228	135	242	571	235	1411
Total	791	635	1353	3222	1507	7508

**Table 5:** Distribution of images across angiographic phases under the Chinese six-grade DR classification system.

Phase Group	Level 1	Level 2	Level 3	Level 4	Level 5	Level 6	Total
V	207	465	942	320	116	51	2101
R	293	646	1709	521	147	117	3433
L	135	242	571	172	40	23	1183
Total	635	1353	3222	1013	303	191	6717

**Table 6:** Distribution of images across angiographic phases for the binary DR classification system after class balancing.

Phase Group	International		China	
	NPDR	PDR	NPDR	PDR
V	488	488	488	488
R	669	669	669	669
L	270	270	270	270

**Table 7:** Performance of the two staging models on FFA images with different angiographic phases under the International five-grade DR classification system.

Model	Phase Group	Accuracy (%)	Precision (%)	Recall (%)	F1-Score (%)
Swin Transformer	V	83.55	82.15	80.12	80.83
	R	81.48	79.94	78.12	78.65
	L	77.46	77.16	76.07	76.44
ConvNeXt	V	86.67	84.62	85.26	84.91
	R	83.84	82.50	80.75	81.25
	L	81.87	80.06	80.60	79.88

**Table 8:** Results of the final generalized linear mixed-effects models with patient ID as a random intercept based on the International five-grade DR classification system.

Model	χ^2^ Phase (*p*)	χ^2^ Eye Side (*p*)	χ^2^ Orientation (*p*)	ICC_ID_	Conditional *R*^2^	Marginal *R*^2^
Swin Transformer	3.173 (0.205)	0.044 (0.834)	4.569 (0.803)	0.786	0.790	0.042
ConvNeXt	5.683 (0.058)	3.250 (0.071)	3.167 (0.923)	0.891	0.896	0.050

**Table 9:** Bonferroni-corrected pairwise comparisons of marginal mean probabilities among FFA phase groups based on the International five-grade DR classification system.

Contrast	Swin Transformer OR (95% CI)	Z	*p*_adj	SMD	ConvNeXt OR (95% CI)	Z	*p*_adj	SMD
V vs. R	0.988 (0.856, 1.142)	−0.209	1.000	−0.001	0.884 (0.719, 1.095)	−1.416	0.470	−0.039
V vs. L	1.076 (0.918, 1.265)	1.107	0.805	0.008	1.044 (0.823, 1.336)	0.436	1.000	0.014
R vs. L	1.090 (0.970, 1.225)	1.762	0.234	0.010	1.181 (0.986, 1.412)	2.209	0.082	0.052

**Table 10:** Performance of the two staging models on FFA images with different angiographic phases under the Chinese six-grade DR classification system.

Model	Phase Group	Accuracy (%)	Precision (%)	Recall (%)	F1-Score (%)
Swin Transformer	V	80.87	86.00	77.37	80.97
	R	79.45	77.01	70.77	73.40
	L	76.66	65.88	61.40	63.13
ConvNeXt	V	85.78	89.57	86.60	87.77
	R	85.53	83.16	80.26	81.56
	L	80.98	77.25	74.29	75.64

**Table 11:** Results of the final generalized linear mixed-effects models with patient ID as a random intercept based on the Chinese six-grade DR classification system.

Model	χ^2^ Phase (*p*)	χ^2^ Eye Side (*p*)	χ^2^ Orientation (*p*)	ICC_ID_	Conditional *R*^2^	Marginal *R*^2^
Swin Transformer	1.044 (0.593)	1.733 (0.188)	7.315 (0.503)	0.744	0.745	0.084
ConvNeXt	2.516 (0.284)	0.500 (0.480)	3.538 (0.896)	0.761	0.768	0.032

**Table 12:** Bonferroni-corrected pairwise comparisons of marginal mean probabilities among FFA phase groups based on the Chinese six-grade DR classification system.

Contrast	Swin Transformer OR (95% CI)	Z	*p*_adj	SMD	ConvNeXt OR (95% CI)	Z	*p*_adj	SMD
V vs. R	1.019 (0.889, 1.170)	0.328	1.000	0.002	0.953 (0.780, 1.167)	−0.574	1.000	−0.015
V vs. L	0.969 (0.830, 1.136)	−0.492	1.000	−0.004	1.063 (0.852, 1.336)	0.662	1.000	0.019
R vs. L	0.951 (0.844, 1.077)	−1.021	0.923	−0.006	1.116 (0.945, 1.323)	1.579	0.343	0.035

**Table 13:** Performance of the two staging models on FFA images with different angiographic phases under the International NPDR vs. PDR classification system.

Model	Phase Group	Accuracy (%)	Precision (%)	Recall (%)	F1-Score (%)
Swin Transformer	V	93.75	91.57	89.26	90.35
	R	93.72	91.37	85.50	88.07
	L	92.76	89.82	85.38	87.36
ConvNeXt	V	94.92	95.17	89.32	91.86
	R	94.73	92.33	88.46	90.24
	L	94.06	91.98	87.78	89.68

**Table 14:** Results of the final generalized linear mixed-effects models with patient ID as a random intercept based on the International NPDR vs. PDR classification system.

Model	χ^2^ Phase (*p*)	χ^2^ Eye Side (*p*)	χ^2^ Orientation (*p*)	ICC_ID_	Conditional *R*^2^	Marginal *R*^2^
Swin Transformer	4.846 (0.089)	0.011 (0.917)	7.144 (0.521)	0.882	0.893	0.091
ConvNeXt	4.736 (0.094)	0.094 (0.759)	4.089 (0.849)	0.964	0.965	0.039

**Table 15:** Bonferroni-corrected pairwise comparisons of marginal mean probabilities among FFA phase groups based on the International NPDR vs. PDR classification system.

Contrast	Swin Transformer OR (95% CI)	Z	*p*_adj	SMD	ConvNeXt OR (95% CI)	Z	*p*_adj	SMD
V vs. R	1.103 (0.968, 1.261)	1.792	0.219	0.005	0.849 (0.696, 1.034)	−1.979	0.143	−0.031
V vs. L	1.018 (0.876, 1.189)	0.283	1.000	0.001	0.828 (0.660, 1.045)	−1.993	0.139	−0.035
R vs. L	0.923 (0.824, 1.032)	−1.686	0.275	−0.004	0.975 (0.818, 1.160)	−0.344	1.000	−0.005

**Table 16:** Performance of the two staging models on FFA images with different angiographic phases under the Chinese NPDR vs. PDR classification system.

Model	Phase Group	Accuracy (%)	Precision (%)	Recall (%)	F1-Score (%)
Swin Transformer	V	93.30	93.41	93.30	93.29
	R	91.73	91.88	91.73	91.72
	L	91.67	91.80	91.67	91.66
ConvNeXt	V	93.30	93.86	93.30	93.28
	R	92.59	92.64	92.59	92.59
	L	91.67	91.68	91.67	91.67

**Table 17:** Results of the final generalized linear mixed-effects models with patient ID as a random intercept based on the Chinese NPDR vs. PDR classification system.

Model	χ^2^ Phase (*p*)	χ^2^ Eye Side (*p*)	χ^2^ Orientation (*p*)	ICC_ID_	Conditional *R*^2^	Marginal *R*^2^
Swin Transformer	2.135 (0.344)	0.033 (0.857)	3.003 (0.934)	0.816	0.817	0.037
ConvNeXt	5.745 (0.566)	0.430 (0.512)	6.667 (0.573)	0.792	0.794	0.063

**Table 18:** Bonferroni-corrected pairwise comparisons of marginal mean probabilities among FFA phase groups based on the Chinese NPDR vs. PDR classification system.

Contrast	Swin Transformer OR (95% CI)	Z	*p*_adj	SMD	ConvNeXt OR (95% CI)	Z	*p*_adj	SMD
V vs. R	1.021 (0.856, 1.218)	0.248	1.000	0.001	0.816 (0.641, 1.045)	−2.026	0.128	−0.065
V vs. L	1.132 (0.913, 1.412)	1.393	0.491	0.010	1.034 (0.762, 1.407)	0.259	1.000	0.011
R vs. L	1.117 (0.907, 1.375)	1.257	0.627	0.008	1.267 (0.945, 1.707)	1.930	0.161	0.076

## Data Availability

The datasets generated and/or analyzed during the current study are not publicly available due to patient privacy and ethical restrictions.

## Supplementary Materials

The following supporting information can be downloaded at: https://www.mdpi.com/article/10.3390/bioengineering13070791/s1.
