# Supplementary material for: Intelligent Staging Performance of Diabetic Retinopathy Based on Fundus Fluorescein Angiography Images with Different Angiographic Phases
Source: Bioengineering (Basel). 2026 Jul 10;13(7):791. doi: 10.3390/bioengineering13070791 (PMC13404394; doi:10.3390/bioengineering13070791)
Supplement: Supplementary file 1 [file bioengineering-13-00791-s001.zip › bioengineering-4366962-supplementary.pdf]

Supplementary Table S1. Model selection results based on AIC and BIC for different random-effects structures across classification tasks and models

System	Model	Random-effects structure	AIC	BIC
International five-grade	Swin Transformer	(1∣Patient ID)	**-1976.0**	**-1903.6**
		(1∣Patient ID/Eye Side)	-1974.0	-1896.4
		(1∣Patient/Eye Side/Fundus Orientation)	-1972.0	-1889.2
	ConvNeXt	(1∣Patient ID)	**-6222.5**	**-6150.0**
		(1∣Patient ID/Eye Side)	-6220.5	-6142.8
		(1∣Patient/Eye Side/Fundus Orientation)	-6218.5	-6135.6
Chinese six-grade	Swin Transformer	(1∣Patient ID)	**-1889.4**	**-1816.7**
		(1∣Patient ID/Eye Side)	-1887.4	-1809.5
		(1∣Patient/Eye Side/Fundus Orientation)	-1885.4	-1802.3
	ConvNeXt	(1∣Patient ID)	**-5340.0**	**-5268.2**
		(1∣Patient ID/Eye Side)	-5338.0	-5261.1
		(1∣Patient/Eye Side/Fundus Orientation)	-5336.0	-5253.9
Binary (International)	Swin Transformer	(1∣Patient ID)	**-3695.9**	**-3623.3**
		(1∣Patient ID/Eye Side)	-3693.9	-3616.1
		(1∣Patient/Eye Side/Fundus Orientation)	-3691.9	-3609.0
	ConvNeXt	(1∣Patient ID)	**-9903.7**	**-9831.2**
		(1∣Patient ID/Eye Side)	-9901.7	-9824.0
		(1∣Patient/Eye Side/Fundus Orientation)	-9899.7	-9816.9
Binary (Chinese)	Swin Transformer	(1∣Patient ID)	**-1379.2**	**-1318.5**
		(1∣Patient ID/Eye Side)	-1377.2	-1312.2
		(1∣Patient/Eye Side/Fundus Orientation)	-1375.2	-1305.8
	ConvNeXt	(1∣Patient ID)	**-4306.5**	**-4246.4**
		(1∣Patient ID/Eye Side)	-4304.5	-4240.1
		(1∣Patient/Eye Side/Fundus Orientation)	-4302.5	-4233.8

Notes: AIC: Akaike Information Criterion; BIC: Bayesian Information Criterion. The random-effects structures are: (1 | Patient ID), (1 | Patient ID/Eye Side), and (1 | Patient ID/Eye Side/Fundus Orientation). The structure with the lowest AIC and BIC values for each model-task combination is highlighted, indicating the final parsimonious model used for subsequent statistical inference.

Supplementary Table S2. Estimated marginal mean response probabilities from the final generalized linear mixed-effects model (GLMM)

System	Model	Venous	Recirculation	Late
International five-grade	Swin Transformer	0.807	0.809	0.796
	ConvNeXt	0.899	0.910	0.895
Chinese six-grade	Swin Transformer	0.791	0.788	0.797
	ConvNeXt	0.905	0.909	0.900
Binary (International)	Swin Transformer	0.906	0.897	0.905
	ConvNeXt	0.950	0.957	0.958
Binary (Chinese)	Swin Transformer	0.891	0.889	0.878
	ConvNeXt	0.937	0.948	0.935

Note: Response probabilities rePrecisionsent estimated marginal means (EMMs) derived from the final generalized linear mixed-effects model with patient ID included as a random intercept.

Supplementary Table S3. Comparison of rePrecisionsentative AI-based DR analysis studies using FFA images

Study	Dataset	Images	Model	Key Metrics	Remarks
**Our Study**	Single-center retrospective data (China); includes different angiographic phases (venous, recirculation, late); annotated according to both International five-grade and Chinese six-grade standards; eye level split.	7,508 (863 eyes)	Swin Transformer; ConvNeXt	Multiclass classification: ConvNeXt best accuracy 86.67% (International five-grade), 85.78% (Chinese six-grade); Binary classification (NPDR vs. PDR): accuracy > 93%; Systematic evaluation of angiographic phase effects on staging performance, with no statistically significant differences by GLMM.	First systematic quantification of angiographic phase effects on model staging performance; rigorous statistical testing with effect size reporting; comparison of Transformer vs. CNN architectures.
**Pan et al. (2020) [21]**	Single-center retrospective data (China); DR patients only; FFA images.	4,067 (221 patients)	DenseNet, ResNet50, VGG16	Multi-label lesion classification: Best model (DenseNet) AUC 0.870–0.965 (for NP, MA, leakage, laser scars, respectively).	First multi-label automatic recognition of multiple DR lesions (non-perfusion, microaneurysms, leakage, laser scars) on FFA images.
**Gao et al. (2022) [20]**	Single-center retrospective data (China); DR patients only; FFA report images.	11,214 (1,114 eyes)	VGG16, ResNet50, DenseNet	Multiclass classification: Best model (VGG16) accuracy 94.17%; external test accuracy 82.47%–88.89%.	Used "nine-square grid input" to integrate multiple FFA images; multi-center validation; provided a benchmark for FFA-based DR grading.
**Zhao et al. (2023) [16]**	Multi-center retrospective data (China); includes DR, BRVO, retinal vasculitis, etc.; FFA images.	24,316 (4,047 eyes)	ResNet-152 (classification); U-Net (segmentation)	Classification: Image phase identification AUC 0.991–0.999; DR/BRVO diagnosis AUC 0.979–0.992; Segmentation: NPA segmentation DSC 83.6%–90.1%; Proposed clinically applicable ischemia index (CAII) to guide laser therapy.	Multi-task system: First one-stop AI system achieving phase identification, multi-disease diagnosis, lesion segmentation, and treatment suggestion from FFA images; strong generalization

Study	Dataset	Images	Model	Key Metrics	Remarks
					capability.
**Chen et al. (2024) [18]**	Single-center retrospective data (China); includes 31 retinal conditions; FFA images paired with Chinese reports.	654,343 (9,392 reports)	BLIP (image-text Precision-training); Llama 2 (LLM)	Report generation: BERTScore 0.70; Top-5 lesion F1 0.64–0.82; Q&A: 70.7% error-free answers, 65.3% satisfied experts.	First generative AI system for FFA report generation and medical question-answering; emphasized human-AI interactive clinical potential.
**Liu et al. (2025) [11]**	Single-center retrospective data (China); DR patients; FFA images.	15,930 (1,593 eyes)	D-GET (Group-Enhanced Transformer)	Grading (4-class): Accuracy 94.66%; Precisioncision 94.69%.	Proposed "16-grid input" method to integrate multi-phase information; used Transformer architecture to improve small lesion detection.

Note: BRVO: Branch Retinal Vein Occlusion; NPA: non-perfusion area; MA: microaneurysms; AUC: area under the curve; DSC: Dice similarity coefficient; GLMM: generalized linear mixed-effects model; BERTScore: a text generation evaluation metric.

Supplementary Table S4. Per-class performance of the Swin Transformer based model on FFA images with different angiographic phases under the International five-grade DR classification system

Phase Group	DR Level	Precision (%)	Recall (%)
Venous (V)	Level1	95.45	80.77
	Level2	71.43	66.67
	Level3	68.89	83.78
	Level4	87.00	87.88
	Level5	88.00	81.48
Recirculation (R)	Level1	79.61	91.67
	Level2	74.60	56.63
	Level3	75.30	78.12
	Level4	84.68	86.54
	Level5	85.51	77.63
Late (L)	Level1	82.50	86.84
	Level2	74.29	60.47
	Level3	67.07	72.37
	Level4	80.50	81.53
	Level5	81.43	79.17

Supplementary Table S5. Per-class performance of the ConvNeXt based model on FFA images with different angiographic phases under the International five-grade DR classification system

Phase Group	DR Level	Precision (%)	Recall (%)
Venous (V)	Level1	88.89	88.89
	Level2	78.95	78.95
	Level3	76.32	82.86
	Level4	90.29	88.57
	Level5	88.68	87.04
Recirculation (R)	Level1	80.13	94.70
	Level2	76.92	60.24
	Level3	77.64	78.12
	Level4	86.56	88.46
	Level5	91.24	82.24
Late (L)	Level1	74.47	92.11
	Level2	78.79	60.47
	Level3	80.26	80.26
	Level4	86.27	84.08
	Level5	80.52	86.11

Supplementary Table S6. Per-class performance of the Swin Transformer based model on FFA images with different angiographic phases under the Chinese six-grade DR classification system

Phase Group	DR Level	Precision (%)	Recall (%)
Venous (V)	Level1	70.83	73.91
	Level2	78.72	68.52
	Level3	80.70	92.00
	Level4	85.71	75.00
	Level5	100.00	71.43
	Level6	100.00	83.33
Recirculation (R)	Level1	78.16	81.93
	Level2	78.21	76.25
	Level3	81.79	87.40
	Level4	74.74	64.55
	Level5	72.22	52.00
	Level6	76.92	62.50
Late (L)	Level1	76.74	76.74
	Level2	73.42	76.32
	Level3	81.10	84.71
	Level4	74.00	67.27
	Level5	50.00	30.00
	Level6	40.00	33.33

Supplementary Table S7. Per-class performance of the ConvNeXt based model on FFA images with different angiographic phases under the Chinese six-grade DR classification system

Phase Group	DR Level	Precision (%)	Recall (%)
Venous (V)	Level1	85.00	94.44
	Level2	94.74	81.82
	Level3	86.27	89.80
	Level4	71.43	73.53
	Level5	100.00	80.00
	Level6	100.00	100.00
Recirculation (R)	Level1	91.03	87.65
	Level2	79.38	86.39
	Level3	89.63	88.35
	Level4	77.66	76.04
	Level5	81.25	76.47
	Level6	80.00	66.67
Late (L)	Level1	86.36	88.37
	Level2	75.34	72.37
	Level3	82.61	84.71
	Level4	83.64	83.64
	Level5	55.56	50.00
	Level6	80.00	66.67

Supplementary Table S8. Per-class performance of the Swin Transformer and ConvNeXt based models on FFA images with different angiographic phases under the International binary (NPDR vs. PDR) classification system

Model	Phase Group	DR Level	Precision (%)	Recall (%)
**Swin Transformer**	Venous (V)	NPDR	95.15	97.03
		PDR	88.00	81.48
	Recirculation (R)	NPDR	94.65	97.97
		PDR	88.10	73.03
	Late (L)	NPDR	94.15	97.14
		PDR	85.48	73.61
**ConvNeXt**	Venous (V)	NPDR	94.79	99.01
		PDR	95.56	79.63
	Recirculation (R)	NPDR	95.77	97.97
		PDR	88.89	78.95
	Late (L)	NPDR	95.06	97.78
		PDR	88.89	77.78

Supplementary Table S9. Per-class performance of the Swin Transformer and ConvNeXt based models on FFA images with different angiographic phases under the Chinese binary (NPDR vs. PDR) classification system

Model	Phase Group	DR Level	Precision (%)	Recall (%)
**Swin Transformer**	Venous (V)	NPDR	95.65	90.72
		PDR	91.18	95.88
	Recirculation (R)	NPDR	94.40	88.72
		PDR	89.36	94.74
	Late (L)	NPDR	89.47	94.44
		PDR	94.12	88.89
**ConvNeXt**	Venous (V)	NPDR	98.84	87.63
		PDR	88.89	98.97
	Recirculation (R)	NPDR	91.27	94.26
		PDR	94.02	90.91
	Late (L)	NPDR	90.91	92.59
		PDR	92.45	90.74
